# Correlation between clinical and pathological features of oral lichen planus: A retrospective observational study

**DOI:** 10.1097/MD.0000000000014614

**Published:** 2019-02-22

**Authors:** Paula Boñar-Alvarez, Mario Pérez Sayáns, Abel Garcia-Garcia, Cintia Chamorro-Petronacci, Pilar Gándara-Vila, Romina Luces-González, Eva Otero Rey, Andres Blanco-Carrión, JM Suárez-Peñaranda

**Affiliations:** a Oral Medicine, Oral Surgery and Implantology Unit, Faculty of Medicine and Dentistry, Santiago de Compostela; b Oral Medicine, Oral Surgery and Implantology Unit, Faculty of Medicine and Dentistry, Instituto de Investigación Sanitaria de Santiago (IDIS); c Department of Pathology, Clinical Hospital, Santiago de Compostela, Spain.

**Keywords:** chronic inflammatory, concomitant medication, oral lichen planus, oral lichenoid lesions

## Abstract

Oral lichen planus (OLP) is a common, chronic, inflammatory disease of autoimmune origin. The aim of this study is to determine the correlation of the histopathological features with clinical aspects and variants of OLP.

We have retrospectively studied a group of 59 adult patients with confirmed clinical and histopathological diagnosis of OLP from the Oral Pathology Unit of the University of Santiago de Compostela (Spain). Clinical parameters: age, gender, location of the lesions, clinical type, toxic habits, and concomitant treatments were evaluated. Histopathologically, the epithelial response (hyperplasia vs atrophy), presence of ulceration, degree of interface lesion and distribution, intensity, and composition of the inflammatory infiltrate were analyzed.

Patients treated with several systemic drugs had more atrophic/erosive forms of OLP (*P* = .019). Plasma cells were found more commonly in cases showing deep inflammatory involvement of the connective subepithelial tissue than in those where inflammation was only superficially located (*P* <.001). Their presence was also associated with epithelial erosion-ulceration (*P* <.01).

In conclusion, patients treated with several drugs had more atrophic/erosive forms of OLP and frequently associated with a deep specific inflammatory pattern based on plasma cells. Our results could suggest that drug intake by some patients might confer a supplementary aggravating character to the disease, alone or in conjunction with other non-identified factors. More studies should be carried out to confirm this trend and to assess whether this characteristic, can actually influence the evolution of the lesions.

## 1 Introduction

Oral lichen planus (OLP) is a common, chronic, inflammatory disease of autoimmune origin. Although epidemiological studies lack clear diagnostic criteria or common methodology, its prevalence is estimated to be between 0.1% and 5% of the general population worldwide (average 1%) and its incidence around 2.2%.^[1]^ It usually presents in females during the fifth and sixth decades of life and only rarely affects children.^[2]^ Its relapsing condition and malignant transformation have been reported very rarely.^[3]^

OLP is a complex disease that can be caused or triggered by genetic malfunction and/or environmental factors. Though etiology remains unknown, an autoimmune basis is generally accepted and several inducers are well known, such as psychological stress, drug intake, hepatitis C virus infection or diabetes.^[2]^ Nevertheless, the precise role of most of them is not well known.

**Figure 1:**
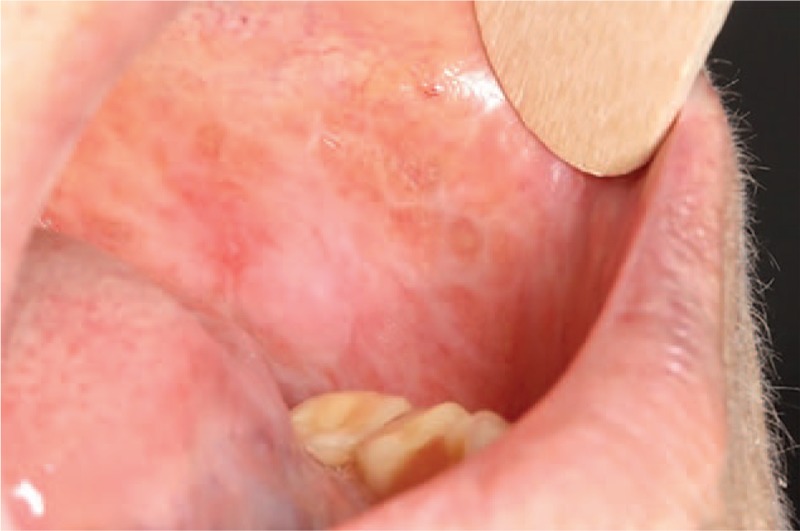
Oral lichen planus, reticular and atrophic lesions.

Clinically, 5 subtypes are usually accepted: reticular, plaque-like, atrophic, erosive-ulcerative, and bullous.^[4]^ For the sake of concision and trying to improve clinic-pathological correlation, they are commonly grouped in 2 categories: those with only reticular lesions and those with atrophic-erosive lesions with or without concomitant reticular lesions (Fig. 1).^[5]^ The most commonly affected oral location in any of the types is the buccal mucosa, usually with symmetrical involvement, followed by the tongue and less frequently the gingiva, lip, and palate.^[6]^

The histopathological features of OLP encompass a spectrum of features, potentially influenced by the stage and activity of the disease at the time of the biopsy, by any recent therapy of the condition. There are contradictory opinions about differences according to the clinical type (reticular vs erosive) and location of the biopsy in the oral cavity. Essentially, it is characterized by a band-like subepithelial lymphocytic infiltrate, presence of intraepithelial lymphocytes with interface lesion.^[1]^ It is usually agreed that oral lichenoid lesions (OLL) are clinically and histologically similar to OLP, but the former lack the characteristic morphology and distribution of OLP.^[7]^ Triggering factors of OLL are usually identifiable and include amalgam fillings, intake of particular drugs or systemic diseases although they can often be idiopathic.^[8]^ Nevertheless, differential diagnosis is of relevance and it may be difficult in many cases, and clinic-pathological correlation is mandatory.^[9]^

In order to improve the distinction between both conditions, a comprehensive knowledge of histopathological features of OLP and its variations with the clinical type of disease and other exogenous factors such as smoking, alcohol intake, and concomitant treatments is mandatory. Nevertheless, few studies have described in detail the clinical and histopathological differences of OLP according to the synchronous presence of the aforementioned factors.

The aim of this study is to determine the correlation of the histopathological features of OLP with the clinical subtypes and other clinical and medical aspects of the patients.

## 2 Material and methods

### 2.1 Study design, setting, and participants

This is a retrospective observational study approved by the local research ethics committee of the University Clinical Hospital of Santiago de Compostela (Ref. 1023/17) and conducted according the STROBE recommendations for observational studies. Data were collected from January 2017 to June 2018 from patients treated in the last 5 years. All procedures were carried out with the adequate understanding and written consent of the subjects in accordance with the Declaration of Helsinki.

Inclusion criteria: patients older than 18 years old with confirmed clinical and histopathological diagnosis of OLP (not OLL) from the Oral Pathology Unit of the University of Santiago de Compostela (Spain). Patients must have at least 2 years of follow-up.

Exclusion criteria: patients younger than 18 years old, patients with no access to their clinical history and OLL with a well-known etiology were excluded

We have evaluated a total of 214 patients with initial suspicious diagnostic of lichen planus and after applying our exclusion criteria, the final sample was 59 patients with OLP.

### 2.2 Variables and data sources

Diagnosis of OLP was achieved using the criteria described by Van der Meij and Van der Waal.^[10]^ In brief, clinically the presence of bilateral and symmetrical lesions with a lace-like network of gray-white lines (reticular pattern), with or without erosive-ulcerative, atrophic, bulbous, or plaque-type lesions, was required for the diagnosis. Histologically, only lesions with a well-defined, band-like, zone of lymphoid infiltration with liquefaction degeneration in the basal cell layer in absence of epithelial dysplasia were considered.

Clinical parameters were retrieved from patient's medical files and data recorded included age, sex, number of lesions, location of the lesions in the oral cavity, clinical type, toxic habits (alcohol and cigarette smoking), OLP management and presence of concomitant treatments as well as other pathological conditions present at the moment of the diagnosis or appearing later in the course of the disease.

Topographically, the lesions were grouped in 5 categories: buccal mucosa, tongue, gingiva, palate or multiple. Clinically, 3 groups were considered: white (predominance of bilateral, symmetrical lesions with white reticular pattern), red (when atrophic or erosive-ulcerative lesions were the most relevant finding), or mixed (cases showing similar proportions of the 2 previous types).

Cigarette smoking was assessed according to the number of cigarettes per day. Daily units of alcohol intake could not be obtained and patients admitting alcohol drinking were stratified by the consumption of low content (<20% volume) or high alcohol content beverages.

Paraffin blocks and stained slides were available for review in all cases. Histopathologically, the epithelial response (hyperplasia vs atrophy), presence of ulceration, degree of interface lesion (defined as follows: mild-only occasional dyskeratotic keratinocytes-; moderate-continuous dyskeratotic keratinocytes along the basal layer- and severe: continuous dyskeratotic keratinocytes along the basal layer forming groups), and distribution (continuous or discontinuous superficial band-like inflammatory infiltrate, and additional presence of perivascular deep inflammatory cells), density and composition of the inflammatory response (lymphocytes, histiocytes, plasma cells, and eosinophils), were evaluated.

### 2.3 Statistical methods

Descriptive statistics were calculated using the frequencies and percentages for the categorical variables and the means and the standard deviations for the quantitative variables. Contingency tables were constructed using the chi-square test. Analytical statistics were performed by comparing means using the 1-way analysis of variance (ANOVA). All the differences in which the value of *P* was less than or equal to .05 were considered statistically significant.

## 3 Results

### 3.1 Clinical and medical aspects

A total of 59 patients (36 women and 23 men) were included in our study, with ages ranging from 30 to 81 years (median 57, interquartile range: 49–57). Clinical aspects and their relations with clinical type of OLP are summarized in Table 1.

**Table 1:**
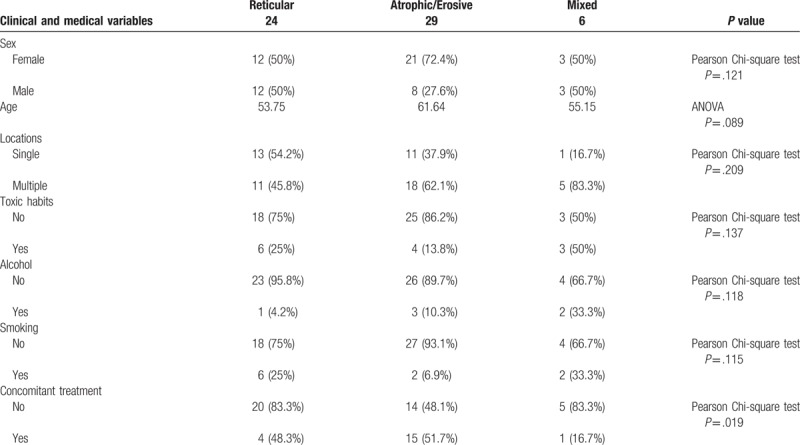
Summary of clinical data.

Erosive-ulcerative and atrophic OLP were considered together under the designation of red OLP and this was the most common clinical variant in our series (49.2%), followed by reticular and mixed forms, with 40.7% and 10.2% of the cases, respectively.

Concomitant medication was being administered to 27 of the 59 patients (45.8%) and included antidepressants or anxiolytic drugs, statins, bisphosphonates, antiaggregants, and antihypertensive drugs. Twenty patients (32.2%) were having several drugs simultaneously and the remaining 54.2% was not under any kind of medical treatment. Patients treated with several drugs had more atrophic/erosive forms of OLP (Pearson Chi-square test, *P* = .019).

Clinical type was not statistically related with age, gender, location, neither toxic habits.

### 3.2 Histopathological aspects

Histopathological aspects and their relations with clinical type of OLP are summarized in Table 2.

**Table 2:**
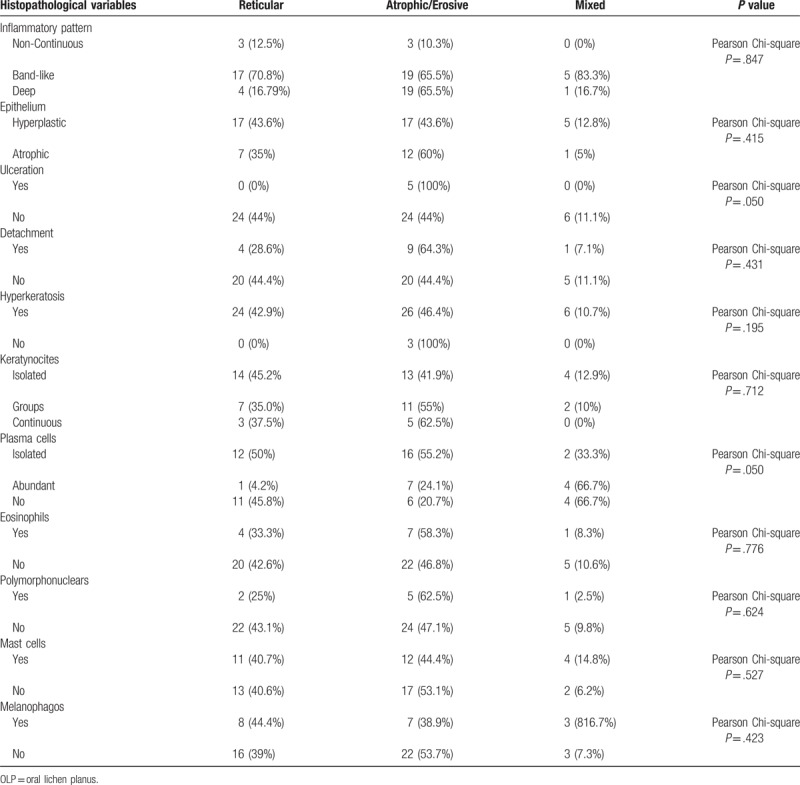
Summary of histopathological aspects of OLP.

We have found a histopathological association between the ulceration of the epithelium surface and the atrophic/erosive clinical type in 100% of cases (Pearson Chi-square, *P* = .050).

In terms of cellularity of the inflammatory infiltrate, it was variable but always characterized by the presence of lymphocytes. Plasma cells were found in 38 patients (64.4%), 79.3% of atrophic/erosive OLP presents these cells, isolated in 55.2% and abundant in 24.1% (Pearson Chi-square, *P* = .050)

In Table 3, we can observe the relation of these plasma cells with the epithelial erosion and the inflammatory pattern. Their presence was associated with epithelial erosion-ulceration (Pearson Chi-square test, *P* <.01). 97.61% of cases showing deep inflammatory involvement of the connective subepithelial tissue, present plasma cells (Pearson Chi-square, *P* <.001).

**Table 3:**
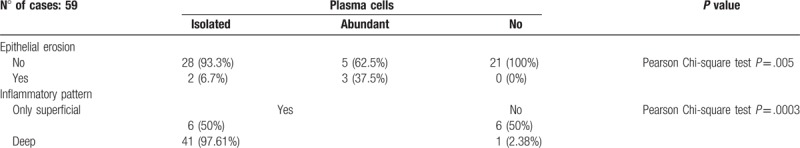
Presence of plasma cells.

Other inflammatory cell types were present, but only in minor proportions, and their presence show no relevant association with clinical or histopathological parameter.

## 4 Discussion

Although difficulties in the clinical diagnosis of OLP are well-known and different degrees of inter observer variability have been reported in the literature,^[10,11]^ adherence to strict criteria is usually enough to rule out other lichenoid mucositis, mainly lichenoid drug reactions. Erosive and atrophic lesions (red lichen planus) were more frequently found than white reticular lesions. If we include mixed forms, they represented 60% of our patients. This is not a constant finding in the literature and discrepancies could be explained by differences in the clinical classification of OLP used, or by a bias in the selection of patients.^[14,15]^ Indeed, our patients have been referred to a specialized unit from different dentists and general practitioners and cases more severe or refractory to treatment might be overrepresented, contributing to the predominance of red lesions.

Women presented with red lesions more commonly than men, as previously reported.^[16–20]^ We have found no explanation for this result since we have not found differences in age and other clinical features between men and women. Conversely, white reticular lesions were more common in patients that admit alcohol intake or cigarette smoking, although was not statistically significant.

A total of 59 patients were included in our study. Median age in our series was 57 years and there was female predominance (1.5:1), coinciding with previous reports in which mean ages were between 41.6 and 59.2 and female to male ratio ranged from 1.32 to 3.29:1.^[4,11]^

In our patients, lesions were more commonly located in the buccal mucosa and in 33.9% was the only location involved by the disease. The tongue and the gingiva followed in frequency, as usually reported in the literature.^[12,13]^ Although location was not related to any clinical or histopathological features.

In relation with this, an additional relevant finding is the association of atrophic or erosive lesions and drug intake. 45.8% of our patients were under systemic treatment, mainly antidepressants and anxiolytic drugs, and 32.3% were having 2 or more drugs simultaneously. These patients seem to present a red form of OLP and the contributing role to aggravate the disease cannot be ruled out. The reason could rest on the multifactorial character of OLP, with patients with coexistent conditions showing a more severe form of disease.^[14,19]^ Alternatively, drugs could trigger an additional inflammatory response that would overlap with OLP and could modify the histopathological aspect of the lesions.^[18]^ Other explanation could lie on the psychosomatic character of some cases of OLP. As noted in previous reports^[17]^ patients with OLP show a higher incidence of psychological disorders than controls and stressing factors might trigger some cases of OLP.^[2]^ This could explain the high incidence of patients treated with psychotropic drugs in our series and could be an aggravating factor for the development of more serious forms of the disease in those patients.

Concerning the cellular composition of the inflammatory response, lymphocytes were the predominant cells in all cases. Plasma cells showed a strong association with those cases with deep extension of the inflammatory process as well as with epithelial erosion, in both cases. The presence of plasma cells in the infiltrate and deep extension around vessels deeply located in the connective tissue are not typical features of OLP and, in fact, they have been recognized as a feature of OLL.^[18,19]^ Even though no clinical or histopathological findings of over-infection were present, we cannot completely rule out subclinical infections as responsible for the presence of plasma cells.

On the other hand, band-like inflammation and interface vacuolar alteration of the basal layer are recognized as key features for the diagnosis of OLP, while other accompanying features, such as parakeratosis, Civatte bodies or fibrinoid deposits along the basement membrane are considered as “non-specific”.^[10,15]^ Though a well-defined lesion in the basal cell layer was present in all our cases, interpreting these cases as OLP is controversial since they fulfill the clinical criteria but show discordant histopathology. They could represent atypical cases of OLP in which morphology has changed by the concomitant action of other factors difficult to assess.^[20]^

Minor differences were present in the pattern of the inflammatory response depending on the clinical type of lesion. Although the continuous band-like pattern was more frequent in white reticular lesions and deep involvement of the deep sub-epithelial connective tissue in red lesions. Previous reports have shown contradictory results about this finding and it has been associated with typical lesions of OLP^[20]^ or considered characteristic of oral lichenoid reactions.^[19]^ Other inflammatory cells were presented but they always represented only a minor part of the inflammatory response and failed to show any association with clinical or histopathological findings.

In summary, we have described that patients treated with several drugs had more atrophic/erosive forms of OLP. According to the position paper of the American Academy of Oral and Maxillofacial Pathology^[1]^ there are no definitive features allowing to differentiate between OLP and oral lichenoid reactions, but our results could suggest that drug intake by some patients might confer a supplementary aggravating character to the disease, alone or in conjunction with other non-identified factors. In terms of cellularity, plasma cells were found more frequently in patients with atrophic/erosive OLP and their presence was associated with epithelial erosion-ulceration and deep inflammatory involvement.

## Author contributions

**Conceptualization:** Paula Boñar-Álvarez.

**Data curation:** Mario Perez Sayans.

**Investigation:** Cintia Chamorro-Petronacci.

**Methodology:** Abel Garcia-García.

**Supervision:** Romina Luces-Gonzalez.

**Validation:** Eva Otero Rey.

**Writing – original draft:** Andres Blanco-Carrion.

**Writing – review & editing:** Pilar Gándara-Vila, JM Suárez-Peñaranda and Mario Pérez-Sayáns.
